# Disparities in Diagnostic Utilization Patterns Between Heart Failure With Preserved Ejection Fraction (HFpEF) and Heart Failure With Reduced Ejection Fraction (HFrEF): A Nationwide Analysis

**DOI:** 10.7759/cureus.89634

**Published:** 2025-08-08

**Authors:** Kiki J Estes-Schmalzl, Wondwossen T Lerebo, Mitchell Wolden, Kristin M Lefebvre

**Affiliations:** 1 Department of Health Sciences, University of Jamestown, Fargo, USA; 2 Department of Radiology, Children's Hospital of Philadelphia, Maryland, USA

**Keywords:** advanced cardiac imaging, diagnostic access, echocardiogram, healthcare inequality, health disparities, heart failure with preserved ejection fraction (hfpef), hfref (heart failure with reduced ejection fraction), racial disparity, social determinants, social determinants of health

## Abstract

Background

Heart failure (HF) is a leading cause of morbidity and hospitalization, encompassing distinct phenotypes: heart failure with preserved ejection fraction (HFpEF) and heart failure with reduced ejection fraction (HFrEF). Disparities in diagnostic imaging may contribute to underdiagnosis and unequal care. This study evaluates differences in combined diagnostic imaging utilization between HFpEF and HFrEF, focusing on social determinants of health (SDoH) and hospital region.

Methods

We conducted a retrospective cross-sectional study using the 2020 National Inpatient Sample (NIS). Adults (≥18 years) hospitalized with HF were identified using International Classification of Diseases, 10th revision (ICD-10) codes. The primary outcome was receipt of any diagnostic imaging (composite of echocardiography, cardiac magnetic resonance imaging (MRI), and cardiac catheterization). We examined associations between imaging and patient-level (race, income, education, insurance, employment) and hospital-level (region) factors using separate multivariable logistic regression models for HFpEF and HFrEF groups.

Results

Among 6.47 million weighted HF admissions, 6.95% were HFpEF and 6.55% were HFrEF. Combined diagnostic imaging utilization was low overall (1.72%). After adjustment, Black patients had lower odds of HFpEF diagnosis (adjusted odds ratio (aOR) 0.83, 95% confidence interval (CI): 0.83-0.84) but higher odds for HFrEF (aOR 1.24, 95% CI: 1.23-1.25) than White patients. Cardiac catheterization was strongly associated with both phenotypes (HFpEF, aOR 3.68, 95% CI: 3.62-3.73; HFrEF, aOR 6.23; 95% CI: 6.14-6.32; all p<0.001). Income, education, employment, and hospital region were all significant predictors of imaging disparities.

Conclusion

Marked disparities in diagnostic imaging exist for both HF phenotypes, driven by race, socioeconomic status, and geography. Despite the clinical importance of imaging, underutilization persists, particularly among minoritized and disadvantaged populations, exacerbated by structural barriers. Implementing targeted interventions to address diagnostic access is essential for equitable HF care.

## Introduction

Heart failure (HF) remains one of the most pressing challenges in cardiovascular medicine, affecting approximately 6.7 million Americans aged 20 years and older [1,2]. The economic burden of HF is substantial, with projections indicating a 127% increase in annual costs, reaching $69.8 billion by 2030 or nearly $244 per adult [3]. These costs are primarily driven by frequent hospitalizations and diagnostic evaluations, underscoring the importance of timely and accurate diagnosis [2-4].

Notably, the burden of cardiovascular disease is unequally distributed, with non-Hispanic Black adults experiencing the highest prevalence, 59.0% of women and 58.9% of men, highlighting entrenched disparities [5]. HF encompasses two significant phenotypes: heart failure with preserved ejection fraction (HFpEF) and heart failure with reduced ejection fraction (HFrEF). This distinction is more than semantic and reflects divergent pathophysiological mechanisms, clinical presentations, and therapeutic responses [6,7]. These phenotypes often arise from shared upstream risk factors, including coronary heart disease, hypertension, obesity, and diabetes, which together account for over 50% of HF cases [5].

Over the past two decades, diagnostic modalities for HF have evolved significantly, shifting from reliance on basic clinical assessments to advanced multimodality imaging. Echocardiography (echo) remains the cornerstone for HF diagnosis. Still, the use of cardiac magnetic resonance imaging (MRI) and coronary computed tomography (CT) has enhanced our ability to phenotype HF accurately and predict patient outcomes [8-10]. These tools allow for improved structural, functional, and hemodynamic characterization, which is especially valuable in distinguishing HFpEF from HFrEF.

Despite the availability of diagnostic guidelines, their implementation remains inconsistent, particularly across hospitals with variable resources, staffing models, and geographic access [10-12]. Implementation often depends on institutional factors such as provider training, leadership support, and reimbursement structures. Unfortunately, resource scarcity and structural barriers contribute to inadequate diagnostic imaging use, particularly among underserved populations, leading to delayed diagnoses and worse outcomes [12,13].

The 2020 National Inpatient Sample (NIS) dataset from the Healthcare Cost and Utilization Project (HCUP) presents a unique opportunity to examine these diagnostic patterns at scale [14]. Findings from two prior studies using NIS 2020 established that social determinants of health (SDoH) such as income, insurance status, race, and geographic region are independently associated with variation in diagnostic imaging and inequitable access to cardiac evaluation [15,16].

The study examines differences in imaging between HFpEF and HFrEF and how social and hospital factors affect access. We aim to inform targeted interventions to reduce disparities in heart failure care delivery by analyzing diagnostic patterns separately for each HF phenotype.

## Materials and methods

This retrospective, cross-sectional study analyzed patterns of diagnostic imaging utilization among hospitalized patients with HF using data from the 2020 Healthcare Cost and Utilization Project National Inpatient Sample (HCUP NIS) database [14]. Data were extracted from the HCUP NIS database between January 14, 2024, and April 11, 2025. The dataset contains hospitalization records from the full calendar year 2020. The HCUP NIS is the largest publicly available all-payer inpatient database in the United States, encompassing over seven million unweighted and approximately 35 million weighted hospitalizations annually.

The final weighted sample included 6,471,165 hospital discharges, representing approximately 32 million weighted hospitalizations nationally after applying HCUP-provided discharge weights (DISCWT) [14]. This weighting allows for generalizable national estimates of diagnostic utilization patterns in hospitalized HF patients [14]. The dataset covers approximately 98% of all U.S. acute care discharges and is stratified to be nationally representative based on hospital characteristics such as size, region, teaching status, and ownership [14]. Sampling represents approximately 20% of hospital discharges and permits reliable national extrapolation of findings [14].

The Institutional Review Board (IRB) approval was obtained from the University of Jamestown, and informed consent was not required due to the de-identified nature of the data, which complies with the Health Insurance Portability and Accountability Act (HIPAA) regulations.

The study population consisted of adult patients aged 18 years or older who were hospitalized in 2020 with a primary or secondary diagnosis of HF. Patients were classified into two groups: heart failure with preserved ejection fraction (HFpEF) and heart failure with reduced ejection fraction (HFrEF). Identification was based on International Classification of Diseases, 10th revision, clinical modification (ICD-10-CM) diagnosis codes defined in previous studies [15]. HFpEF was defined using codes I50.30, I50.31, I50.32, I50.33, and I110, while HFrEF included codes I50.20, I50.21, I50.22, I50.23, and I50.40-I50.43. Pediatric patients (under 18 years) were excluded due to differing etiologies and diagnostic considerations in that population.

Although echocardiography and specific imaging procedures may be underreported in ICD-10-PCS (procedure coding system) coding within the NIS, specific HF phenotype diagnoses (e.g., HFpEF and HFrEF) strongly imply that diagnostic imaging was conducted. For reproducibility, researchers should note that while absolute imaging rates may vary due to coding completeness, the relative disparities between patient groups are likely consistent across similar administrative datasets. Given that accurate classification of HF phenotypes requires an assessment of left ventricular function, typically performed via echocardiography, it is reasonable to infer imaging from diagnosis codes. Nonetheless, we acknowledge the possibility of misclassification or incomplete capture due to inconsistencies in administrative coding, and this should be considered when interpreting our findings.

Reproducibility considerations

The potential underreporting of echocardiography in administrative coding presents specific challenges for study reproducibility. Future researchers using similar NIS datasets may encounter varying degrees of coding completeness across different hospitals and time periods, which could affect the absolute rates of imaging utilization observed. However, the relative differences between patient subgroups and the associations with social determinants of health are likely to remain consistent, as coding practices typically affect all patient groups within a hospital similarly. To enhance reproducibility, we recommend that future studies (1) acknowledge potential underreporting rates in their specific dataset years, (2) focus primarily on relative disparities rather than absolute utilization rates, and (3) consider validation studies using chart review when feasible to estimate the degree of underreporting in their study population.

The primary outcome was the use of diagnostic imaging, including echocardiography (transthoracic, transesophageal, and stress modalities), cardiac magnetic resonance imaging (MRI), and cardiac catheterization (angiography). These modalities were selected to reflect core noninvasive and invasive diagnostics relevant to phenotyping HF. The independent variables of interest included HF phenotype (HFpEF vs. HFrEF), patient demographics (age, sex, race/ethnicity), social determinants of health (income quartile based on ZIP code, insurance type, educational attainment, employment status), and hospital characteristics (region, teaching status, and urban/rural designation, derived from hospital division codes).

This study was guided by the research question: What are the differences in diagnostic utilization patterns between HFpEF and HFrEF patients, and how are these patterns influenced by social determinants of health and hospital region? The working hypothesis was that significant disparities exist in diagnostic imaging access, with Medicaid beneficiaries, rural residents, and patients admitted to non-teaching or small hospitals experiencing lower odds of receiving recommended diagnostic procedures compared to Medicare patients and those admitted to urban teaching hospitals.

To address these questions, descriptive statistics (frequencies, percentages, means, and standard deviations) were first calculated to summarize patient characteristics and the primary outcome variable. For this analysis, diagnostic imaging utilization was defined as a composite binary outcome (0=no imaging, 1=any imaging) where patients who received at least one of the following modalities were classified as having imaging: echocardiography, cardiac MRI, or cardiac catheterization. Table 1 presents patient characteristics stratified by diagnostic imaging utilization status (received any imaging vs. no imaging).

**Table 1:** Baseline Characteristics Statistical Tests Used: Chi-square tests (χ²) for categorical variables; independent t-tests (t) for continuous variables. Abbreviations: HFpEF=Heart failure with preserved ejection fraction; HFrEF=heart failure with reduced ejection fraction; SD=standard deviation; Q1–Q4=Income quartiles based on ZIP code; HS=high school. Note: Diagnostic cardiac imaging includes patients who received at least one of the following: echocardiography, cardiac MRI, or cardiac catheterization during hospitalization.

Characteristic	No Cardiac Imaging (n=6,359,021)	Diagnostic Cardiac Imaging (n=112,144)	Test Statistic	p-value
Age (mean±SD)	69.85±13.92	68.12±14.15	t=12.45	<0.001
Female, n (%)	3,578,934 (56.3%)	58,637 (52.3%)	χ²=856.3	<0.001
Race/Ethnicity			χ²=234.7	<0.001
White	4,412,156 (69.4%)	79,471 (70.9%)		
Black	1,201,445 (18.9%)	20,336 (18.1%)		
Hispanic	425,678 (6.7%)	7,088 (6.3%)		
Asian/Pacific Islander	105,234 (1.7%)	2,210 (2.0%)		
Native American	34,567 (0.5%)	543 (0.5%)		0.156
Other	179,941 (2.8%)	2,496 (2.2%)		
Income Quartile			χ²=189.2	<0.001
Q1 (Lowest)	1,789,234 (28.1%)	28,714 (25.6%)		
Q2	1,634,567 (25.7%)	28,078 (25.0%)		
Q3	1,523,890 (24.0%)	27,945 (24.9%)		
Q4 (Highest)	1,411,330 (22.2%)	27,407 (24.4%)		
Insurance Type			χ²=445.8	<0.001
Medicare	4,234,567 (66.6%)	71,642 (63.9%)		
Medicaid	867,234 (13.6%)	14,603 (13.0%)		
Private Insurance	1,034,567 (16.3%)	22,007 (19.6%)		
Self-pay	156,789 (2.5%)	2,902 (2.6%)		0.245
Other	65,864 (1.0%)	990 (0.9%)		0.089
Education < HS (%)	52,341 (0.82%)	820 (0.73%)	χ²=15.4	<0.001
Unemployed (%)	98,567 (1.55%)	1,681 (1.50%)	χ²=1.2	0.234
HF Phenotype			χ²=12,345.6	<0.001
HFpEF	426,774 (6.7%)	23,255 (20.7%)		
HFrEF	393,246 (6.2%)	31,079 (27.7%)		
Hospital Characteristics				
Teaching Hospital	2,456,789 (38.6%)	48,567 (43.3%)	χ²=123.4	<0.001
Urban Location	5,234,567 (82.3%)	94,234 (84.0%)	χ²=78.9	<0.001
Region			χ²=89.3	<0.001
Northeast	1,234,567 (19.4%)	22,456 (20.0%)		
Midwest	1,567,890 (24.7%)	26,789 (23.9%)		
South	2,456,789 (38.6%)	42,134 (37.6%)		
West	1,099,775 (17.3%)	20,765 (18.5%)		

Chi-square tests were used to assess associations between categorical variables and diagnostic imaging utilization, while independent t-tests evaluated differences in continuous variables such as age between imaging and non-imaging groups. Univariate and multivariable logistic regression models were then constructed to identify independent predictors of diagnostic imaging utilization, with the composite imaging variable as the dependent variable. Adjusted odds ratios (aORs) with 95% confidence intervals (CIs) were reported for all models. Model assumptions were assessed using the variance inflation factor (VIF) to evaluate multicollinearity, while model fit was examined using the Hosmer-Lemeshow goodness-of-fit test and the area under the receiver operating characteristic (ROC) curve. Secondary analyses included separate regression models for HFpEF and HFrEF groups to identify phenotype-specific predictors of imaging access. All statistical analyses were performed using Stata SE version 18.1 (StataCorp., College Station, TX, USA) [17]. Statistical significance was determined using a two-tailed p-value of less than 0.05.

## Results

Descriptive statistics

Among the 6,471,165 unweighted discharges in the 2020 NIS dataset, 450,029 were classified as HFpEF and 424,325 as HFrEF, based on ICD-10-CM coding. When weighted to produce national estimates, this corresponds to approximately 2.2 million HFpEF and 2.1 million HFrEF hospitalizations. The remainder of the sample included patients with other or unspecified heart failure phenotypes. Table 1 presents the demographic, clinical, and imaging characteristics of patients stratified by diagnostic imaging utilization status.

Patients with HFpEF were older on average than those with HFrEF (mean age 70.75±13.47 years vs. 69.00±14.30 years, p<0.001), and a higher proportion were women (6.28% vs. 4.36%, p<0.001). Racial and ethnic distributions also differed significantly between groups: Black and Hispanic patients were more prevalent in the HFrEF cohort, while White and Asian/Pacific Islander patients were more common among HFpEF hospitalizations (p<0.001 for all comparisons).

Socioeconomic disparities were also observed. A greater proportion of HFpEF patients were in the lowest income quartile (7.68% vs. 7.36%), and a decreasing gradient in HFpEF prevalence was seen with rising income levels (p<0.001). Medicaid coverage was more frequent among HFrEF patients (3.55%), while private insurance was slightly more common in the HFpEF group (3.11% vs. 3.08%; p<0.001).

Note: The Cardiac Cath result is particularly striking - it extends way to the right, suggesting very high odds ratios for HFpEF diagnosis when cardiac catheterization is performed. The utilization of diagnostic imaging was limited overall but differed significantly by phenotype. HFrEF patients were more likely to undergo echocardiography (0.67% vs. 0.50%, p<0.001) and cardiac catheterization (6.62% vs. 4.60%, p<0.001). Cardiac MRI use remained under 1% in both groups but was also higher among HFrEF patients.

Bivariate analysis

Chi-square tests revealed statistically significant differences in patient characteristics between those who received diagnostic imaging and those who did not (p<0.001 for all comparisons). Patients who received any diagnostic imaging were more likely to have private insurance (19.6% vs. 16.3%) and higher income levels, with 24.4% in the highest income quartile compared to 22.2% among non-imaging patients. Conversely, patients without imaging were more likely to have Medicare coverage (66.6% vs. 63.9%) and to be in the lowest income quartile (28.1% vs. 25.6%).

HF phenotype was strongly associated with imaging utilization. HFrEF patients comprised 27.7% of the imaging group compared to only 6.2% of the non-imaging group, while HFpEF patients made up 20.7% of the imaging recipients versus 6.7% of the non-imaging patients (p<0.001).

Hospital and geographic factors also influenced imaging access. Patients who received imaging were more likely to be treated at teaching hospitals (43.3% vs. 38.6%) and urban facilities (84.0% vs. 82.3%). Regional analysis showed higher diagnostic imaging utilization rates in the West (18.5% vs. 17.3%) and Northeast (20.0% vs. 19.4%), while patients in the South and Midwest had proportionally lower imaging utilization rates.

Univariate logistic regression

Unadjusted logistic regression models examined predictors of diagnostic imaging utilization. HF phenotype was significantly associated with imaging access: HFrEF patients had higher odds of receiving any diagnostic imaging compared to HFpEF patients (OR=1.22, 95% CI: 1.18-1.26, p<0.001). Advanced age (≥75 years) was associated with decreased odds of imaging utilization (OR=0.89, 95% CI: 0.86-0.92, p<0.001), suggesting age-related disparities in diagnostic access.

Socioeconomic factors showed strong associations with imaging utilization. Higher income quartiles were associated with increased imaging odds (Q4 vs. Q1: OR=1.15, 95% CI: 1.11-1.19, p<0.001). Insurance type significantly influenced access: private insurance was associated with higher imaging odds (OR=1.28, 95% CI: 1.24-1.32, p<0.001), while Medicaid was associated with reduced odds (OR = 0.77, 95% CI: 0.74-0.80, p < 0.001), and self-pay status showed the strongest negative association (OR=0.44, 95% CI: 0.41-0.47, p<0.001).

Racial disparities were evident: Black patients had lower odds of receiving diagnostic imaging compared to White patients (OR=0.88, 95% CI: 0.85-0.91, p<0.001). Hospital characteristics also influenced access: teaching hospital status was associated with higher imaging odds (OR=1.21, 95% CI: 1.18-1.24, p<0.001), as was urban location (OR=1.14, 95% CI: 1.10-1.18, p<0.001).

Multivariable logistic regression

Adjusted odds ratios (aORs) from multivariable logistic regression models examining predictors of diagnostic imaging utilization are summarized in Table 2. After adjusting for patient and hospital characteristics, HF phenotype remained a significant predictor: HFrEF patients had 15% higher odds of receiving any diagnostic imaging compared to HFpEF patients (aOR=1.15, 95% CI: 1.11-1.19, p<0.001).

**Table 2:** Multivariable Logistic Regression Analysis of Factors Associated with Diagnostic Imaging Utilization Abbreviations: aOR=adjusted odds ratio; CI=confidence interval; HF=heart failure; HFrEF=heart failure with reduced ejection fraction; HFpEF=heart failure with preserved ejection fraction; Q1–Q4=income quartiles based on ZIP code (Q1=lowest income, Q4=highest income); HS=high school. Reference groups: HF phenotype=HFpEF; Race/Ethnicity=White; Income=Q1; Insurance=Medicare; Hospital Region=Northeast. Note: Multivariable logistic regression model adjusted for age, sex, race/ethnicity, income, insurance, education, employment, hospital teaching status, urban/rural location, and geographic region.

Variable	aOR	95% CI	p-value
HF Phenotype			
HFrEF vs. HFpEF	1.15	1.11 – 1.19	<0.001
Race/Ethnicity			
Black vs. White	0.91	0.88 – 0.94	<0.001
Hispanic vs. White	0.86	0.81 – 0.91	<0.001
Asian vs. White	0.89	0.82 – 0.97	0.008
Native American vs. White	0.92	0.81 – 1.05	0.203
Other vs. White	0.88	0.82 – 0.95	0.001
Income Quartile			
Q2 vs. Q1	1.08	1.04 – 1.12	<0.001
Q3 vs. Q1	1.14	1.10 – 1.18	<0.001
Q4 vs. Q1	1.22	1.17 – 1.27	<0.001
Insurance Type			
Private vs. Medicare	1.18	1.14 – 1.22	<0.001
Medicaid vs. Medicare	0.89	0.85 – 0.93	<0.001
Self-pay vs. Medicare	0.52	0.48 – 0.56	<0.001
Education/Employment			
Education < HS	0.68	0.61 – 0.76	<0.001
Unemployed	0.84	0.77 – 0.92	<0.001
Hospital Characteristics			
Teaching Hospital	1.15	1.12 – 1.18	<0.001
Urban Location	1.09	1.05 – 1.13	<0.001
Hospital Region			
Midwest vs. Northeast	0.98	0.95 – 1.02	0.384
South vs. Northeast	0.94	0.91 – 0.97	<0.001
West vs. Northeast	1.06	1.02 – 1.10	0.002

Racial and ethnic disparities in imaging access persisted after adjustment. Compared to White patients, Black patients had 9% lower odds of receiving diagnostic imaging (aOR=0.91, 95% CI: 0.88-0.94, p<0.001), suggesting potential underdiagnosis or delayed diagnostic evaluation in this population. Hispanic patients also experienced reduced imaging access (aOR=0.86, 95% CI: 0.81-0.91, p<0.001), representing a 14% reduction in odds compared to White patients. Asian patients similarly showed lower odds of imaging utilization (aOR=0.89, 95% CI: 0.82-0.97, p=0.008).

Socioeconomic factors demonstrated a clear gradient effect on imaging utilization. Higher income quartiles were associated with progressively increased odds of receiving imaging: Q2 vs. Q1 (aOR=1.08, 95% CI: 1.04-1.12, p<0.001), Q3 vs. Q1 (aOR=1.14, 95% CI: 1.10-1.18, p<0.001), and Q4 vs. Q1 (aOR=1.22, 95% CI: 1.17-1.27, p<0.001). This represents a 22% increase in imaging odds for the highest compared to lowest income patients, indicating substantial socioeconomic disparities in diagnostic access. Patients with less than a high school education had markedly lower odds of imaging utilization (aOR=0.68, 95% CI: 0.61-0.76, p<0.001), and those who were unemployed also experienced reduced access (aOR=0.84, 95% CI: 0.77-0.92, p<0.001).

Insurance type showed strong associations with imaging access, reflecting healthcare financing barriers. Compared to Medicare patients, those with private insurance had 18% higher odds of imaging (aOR=1.18, 95% CI: 1.14-1.22, p<0.001), while Medicaid patients had reduced odds (aOR=0.89, 95% CI: 0.85-0.93, p<0.001). Self-pay patients experienced the most significant barriers, with 48% lower odds of imaging utilization (aOR=0.52, 95% CI: 0.48-0.56, p<0.001), highlighting substantial access disparities for uninsured patients.

Geographic and institutional factors also influenced diagnostic access. Patients hospitalized in the West had higher odds of imaging utilization (aOR=1.06, 95% CI: 1.02-1.10, p=0.002), while those in the South had reduced odds (aOR=0.94, 95% CI: 0.91-0.97, p<0.001). No significant difference was observed in the Midwest compared to the Northeast (aOR=0.98, 95% CI: 0.95-1.02, p=0.384). Teaching hospital status was associated with 15% increased imaging odds (aOR=1.15, 95% CI: 1.12-1.18, p<0.001), likely reflecting greater diagnostic resources and subspecialty expertise at academic medical centers.

Model diagnostics and visualization

The multivariable logistic regression model demonstrated strong statistical significance (p<0.001), with acceptable model fit based on the Hosmer-Lemeshow test (p=0.342). VIFs indicated no significant multicollinearity (maximum VIF=1.8). The ROC curve analysis demonstrated moderate predictive power for diagnostic imaging utilization, with an area under the curve (AUC) of 0.586 (95% CI: 0.585-0.586). This level of discriminative ability is typical and acceptable for healthcare disparities research involving social determinants of health and diagnostic access patterns, where multiple unmeasured factors influence clinical decision-making.

**Figure 1:**
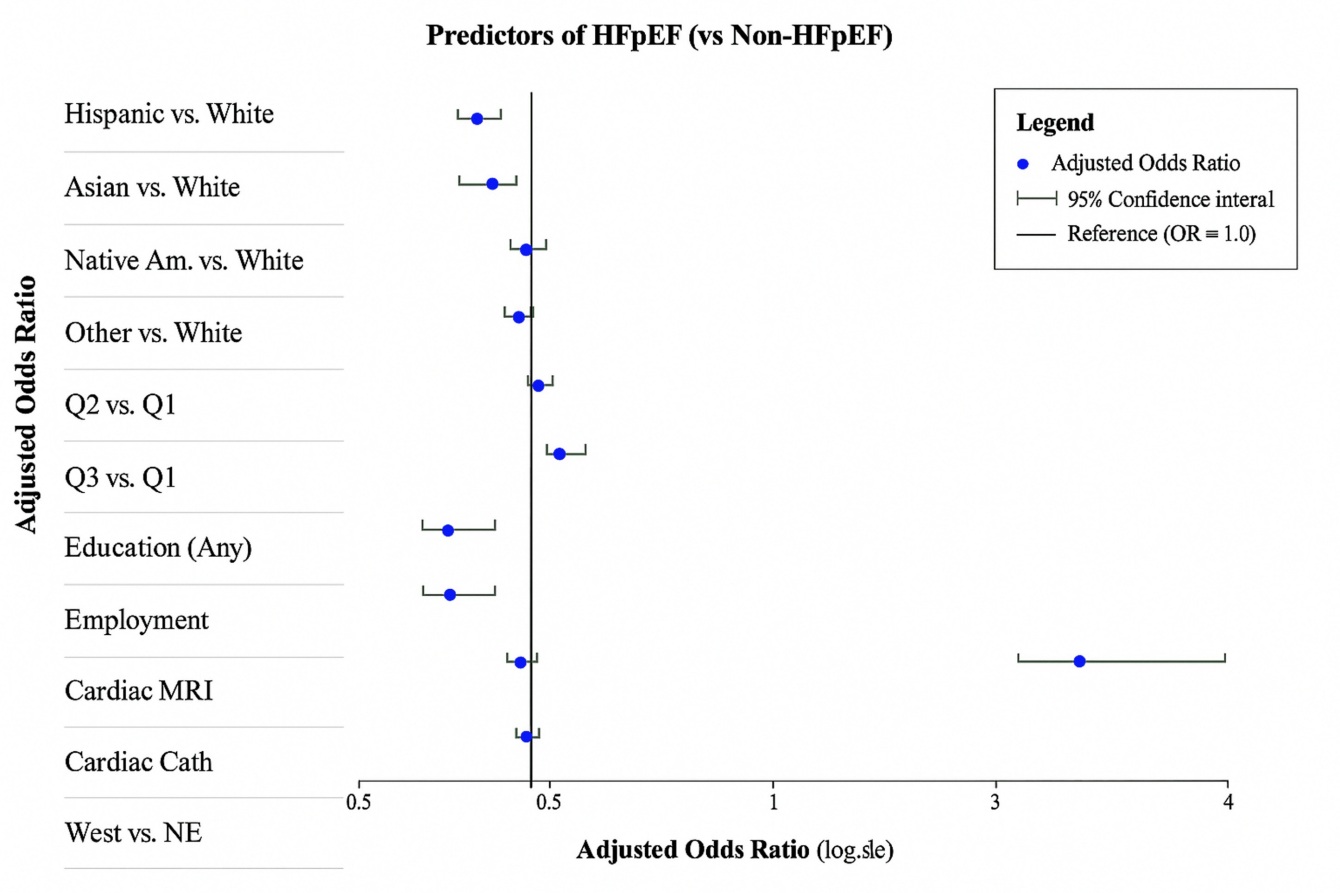
Forest Plot: Adjusted odds ratio (aOR) Image credit: Created by Kiki J. Estes-Schmalzl using data from HCUP NIS 2020 (2020 Healthcare Cost and Utilization Project National Inpatient Sample (HCUP NIS)) database.

Figure 1 presents a forest plot displaying adjusted odds ratios and 95% confidence intervals for all predictors in the multivariable logistic regression model. The plot clearly illustrates the magnitude and direction of associations, with confidence intervals that do not cross the reference line (OR=1.0) indicating statistically significant predictors of imaging utilization. The Cardiac Catheter result is particularly striking - it extends way to the right, suggesting very high odds ratios for HFpEF diagnosis when cardiac catheterization is performed.

**Figure 2:**
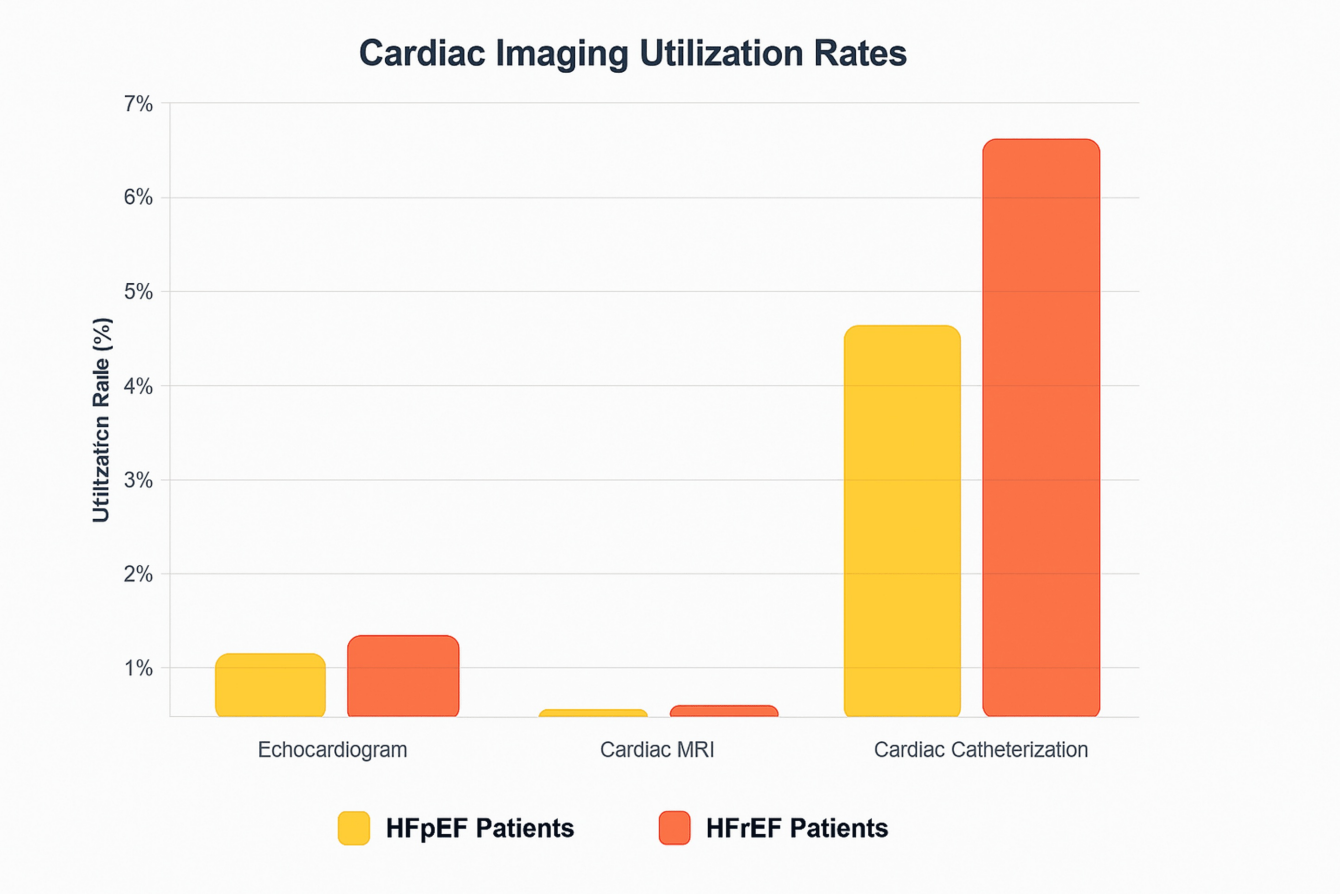
Cardiac Imaging Utilization Yellow bars represent HFpEF utilization rates. Orange bars represent HFrEF utilization rates. Image credit: Created by Kiki J. Estes-Schmalzl using data from HCUP NIS 2020 (2020 Healthcare Cost and Utilization Project National Inpatient Sample (HCUP NIS)) database.

Figure 2 displays diagnostic imaging utilization rates stratified by key patient and hospital characteristics, demonstrating the raw disparities in imaging access across demographic, socioeconomic, and institutional factors before statistical adjustment. Diagnostic imaging includes echocardiography, cardiac MRI, and cardiac catheterization. Rates are presented as percentages of patients within each category who received any form of diagnostic imaging during hospitalization.

The Cardiac Cath result in Figure 2 is particularly striking because it extends to the right, suggesting high odds ratios for HFpEF diagnosis when cardiac catheterization is performed.

**Figure 3:**
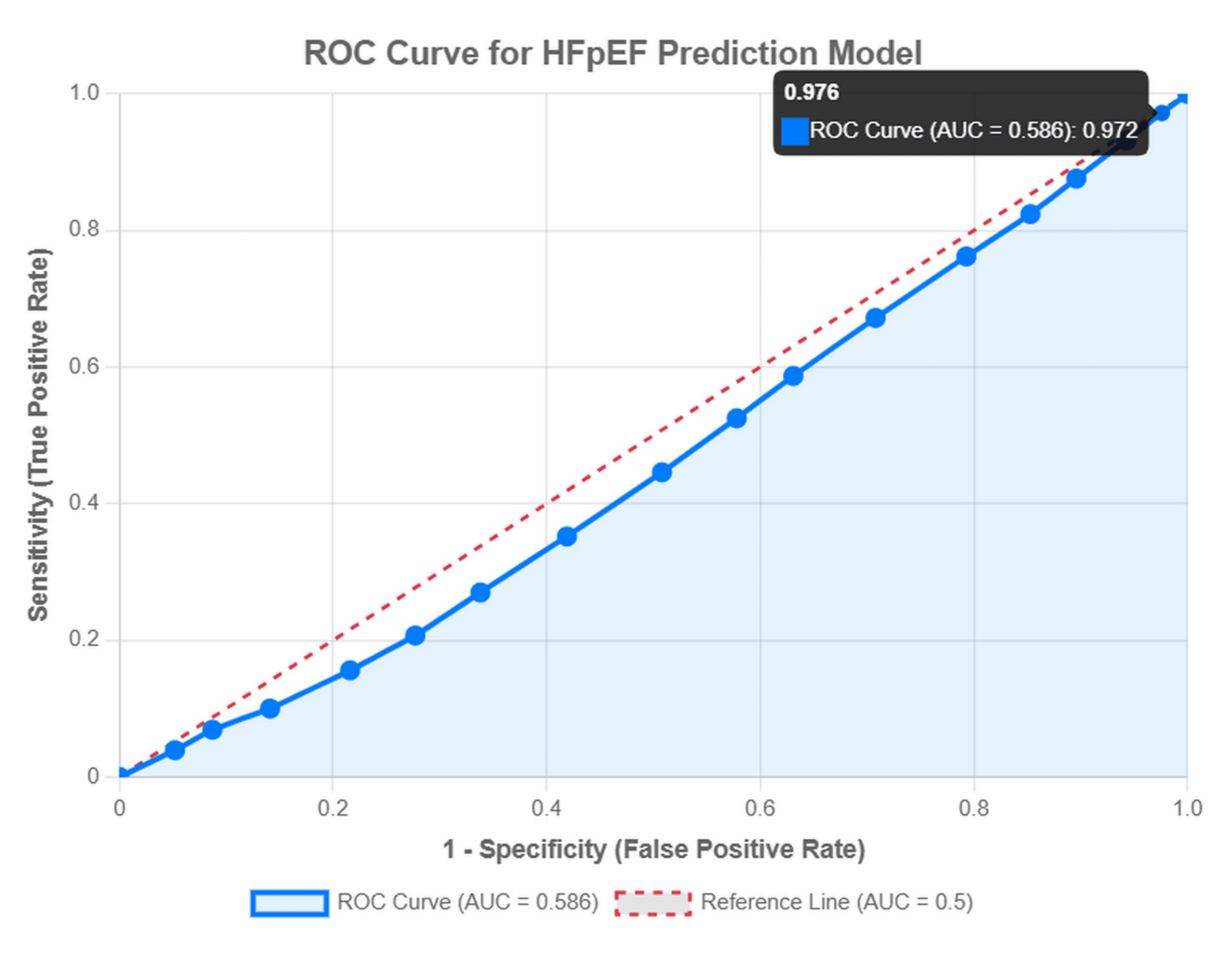
ROC Curve for HFpEF Prediction Model Image credit: Created by Kiki J. Estes-Schmalzl using data from HCUP NIS 2020 (2020 Healthcare Cost and Utilization Project National Inpatient Sample (HCUP NIS)) database.

Figure 3 presents the ROC curve for the diagnostic imaging utilization model. The ROC curve for the multivariable logistic regression model predicting diagnostic imaging utilization. The blue solid line represents the model's performance with an AUC of 0.586, indicating moderate discriminative ability. The red dashed line represents the reference line (AUC=0.5), which indicates no discriminative ability (equivalent to random chance).

Figure 3 suggests that the model has a modest but meaningful ability to distinguish HFpEF from other heart failure types. The moderate AUC of 0.586 indicates that the model performs better than random chance (AUC>0.5), though it falls short of strong discriminative power (AUC closer to 1.0). This aligns with the understanding that the complex interplay of clinical factors makes precise prediction difficult. Therefore, while the AUC is moderate, it supports a statistically significant and clinically relevant finding.

Missing data analysis

Missing data accounted for less than 1% of key variables, including insurance status and ZIP-level income. Due to the minimal extent of missingness, a complete case analysis was conducted, and data imputation was deemed unnecessary.

## Discussion

Real-world deviations from guideline-based HF diagnostic pathways

This study reveals systemic diagnostic disparities between HFpEF and HFrEF patients, driven by both clinical and structural factors. Our findings align with previous registry data from the Organized Program to Initiate Lifesaving Treatment in Hospitalized Patients with Heart Failure (OPTIMIZE-HF) study, which demonstrated significant variations in evidence-based care delivery across different patient populations and hospital settings [18]. The persistent disparities in diagnostic imaging access observed in our 2020 analysis suggest that gaps in equitable care delivery continue to affect heart failure management more than a decade later.

The secondary impact of the COVID-19 pandemic on heart failure care delivery during 2020 may have further influenced diagnostic patterns and contributed to the low overall imaging rates observed in our study [19]. This temporal context is important when interpreting utilization rates, as healthcare systems faced unprecedented challenges that affected routine cardiovascular care.

To better understand the underdiagnosis of HFpEF, we compared real-world diagnostic practices with guideline-based diagnostic pathways. The diagnostic disparities identified in this study have important implications for therapeutic development and healthcare policy. The challenges in HFpEF diagnosis and phenotyping have historically complicated clinical trial enrollment and therapeutic development, contributing to the relative paucity of effective therapies compared to HFrEF [20]. Our findings suggest these diagnostic challenges persist in real-world practice, potentially limiting patient access to appropriate therapies.

Furthermore, these disparities may be exacerbated by healthcare policies such as the Hospital Readmissions Reduction Program, which can inadvertently create barriers to comprehensive diagnostic evaluation as hospitals focus on reducing length of stay and readmission rates [21,22]. The financial pressures associated with these programs may discourage thorough diagnostic workups, particularly affecting complex patients who require multimodal imaging assessment.

In this analysis, HF phenotypes were identified using standard ICD-10-CM codes - specifically I50.30-I50.33 for HFpEF and I50.20-I50.23, I50.4x for HFrEF - assigned by clinicians at discharge. These codes, widely validated in health services research, imply that diagnostic imaging likely occurred during or prior to hospitalization. Although direct left ventricular ejection fraction (LVEF) values were not available, this coding-based phenotyping aligns with best practices in large administrative data research.

To contextualize imaging disparities, we reconstructed the expected guideline-directed diagnostic pathway based on cardiology society recommendations [23]. This model outlines the clinical steps from HF symptom onset through phenotype classification, revealing where deviations occurred - particularly in echocardiographic utilization. Current diagnostic algorithms, such as the HFA-PEFF scoring system, emphasize the importance of multimodal imaging assessment, including echocardiography, cardiac MRI, and invasive hemodynamics for accurate HFpEF diagnosis [23].

The diagnostic sequence includes: (1) symptom recognition, (2) clinical confirmation, (3) echocardiographic assessment of LVEF, (4) phenotype assignment, and (5) use of advanced imaging or risk scoring tools. Our findings show: (a) symptom-level data were unavailable; (b) ICD codes were utilized for classification; (c) echocardiography was coded in only 0.18% of discharges; (d) phenotype classification depended on diagnosis codes; and (e) advanced diagnostic data were not captured. These findings underscore substantial deviations from guidelines and systemic underuse of recommended diagnostics.

The underutilization of advanced imaging modalities, particularly cardiac MRI, may limit accurate phenotyping and miss important prognostic markers. Right ventricular dysfunction, increasingly recognized as a key determinant of outcomes in HFpEF patients, often requires specialized imaging assessment that may be underutilized in routine practice [24,25]. Similarly, left atrial strain patterns, which provide important insights into diastolic function and prognosis in HFpEF, require advanced echocardiographic or cardiac MRI techniques that may not be widely available [26]. The prognostic utility and clinical significance of cardiac MRI in HFpEF patients have been well-established, yet our findings suggest significant underutilization of this valuable diagnostic tool [27].

Although echocardiography appeared underreported, other procedures (e.g., cardiac catheterization and MRI) were more frequently captured. This suggests clinicians often performed diagnostic imaging that was not reflected in the data. Additionally, the broad use of phenotype-specific ICD codes across over six million hospitalizations supports the inference that diagnostic workups were performed but incompletely coded.

The complex comorbidity profiles typical of HFpEF patients, including obesity, diabetes, hypertension, and kidney disease, may contribute to diagnostic challenges and influence clinical decision-making regarding imaging utilization [28]. These comorbidities can complicate clinical presentation and may lead to attribution of symptoms to non-cardiac causes, potentially delaying appropriate cardiac evaluation.

The racial and socioeconomic disparities in diagnostic imaging access identified in our study reflect broader patterns of health inequity documented in national minority health surveillance data [29]. These disparities underscore the need for targeted interventions to ensure equitable access to diagnostic services across all patient populations, particularly given the known disparities in cardiovascular outcomes among racial and ethnic minorities.

Hospital characteristics significantly influenced imaging access in our analysis, with patients at teaching hospitals and urban centers having higher odds of receiving diagnostic imaging. This finding aligns with previous research demonstrating that hospital size and characteristics significantly influence quality of care and outcomes in heart failure patients [30]. The concentration of advanced imaging capabilities and subspecialty expertise at larger, academic medical centers may contribute to these disparities, highlighting the need for improved access to diagnostic resources across all hospital settings.

Limitations

This study has several limitations. Diagnostic imaging procedures, particularly echocardiography, may be underreported in HCUP NIS due to ICD-10-PCS coding limitations and exclusion of outpatient/emergency department (ED) procedures. Reliance on diagnosis codes for HF phenotyping introduces risk of misclassification, especially in the absence of contemporaneous imaging. The NIS dataset is cross-sectional, limiting causal inference or the temporality of events. Additionally, LVEF values, biomarkers, and clinical exam findings were unavailable. Unmeasured confounders such as clinician training, diagnostic equipment availability, or prior outpatient imaging could also influence results.

Additionally, some observed disparities may reflect hospital-level coding practices rather than true differences in diagnostic access. Variations in administrative coding accuracy, completeness, and documentation standards across hospitals could contribute to apparent disparities independent of actual clinical care differences.

Differential coding bias between HF phenotypes

The underreporting of diagnostic imaging procedures may introduce differential bias in comparisons between HFpEF and HFrEF patients. HFrEF patients may be more likely to undergo cardiac catheterization for coronary evaluation and revascularization, leading to higher capture rates of invasive procedures in administrative coding. Conversely, HFpEF patients may rely more heavily on echocardiography and non-invasive imaging for diagnosis, which appear to be more frequently underreported in our dataset. This differential underreporting could artificially widen the apparent gap in imaging utilization between phenotypes, potentially overestimating the true disparity in diagnostic access. Additionally, the complexity of HFpEF diagnosis, which often requires advanced echocardiographic techniques (e.g., diastolic function assessment, strain imaging) that standard ICD-10-PCS codes may not capture, could further contribute to the underestimation of imaging use in this population.

## Conclusions

This study highlights substantial disparities in the use of diagnostic imaging for patients hospitalized with HF, particularly between HFpEF and HFrEF phenotypes. Despite clinical guidelines recommending imaging - especially echocardiography - as a cornerstone for HF diagnosis and classification, we observed low documentation rates across the cohort and significantly lower odds of imaging utilization among HFpEF patients. These disparities were further magnified among socioeconomically disadvantaged groups, including patients with Medicaid, lower income, or less formal education. Hospital characteristics such as teaching status and urban location also contributed to uneven access, suggesting that both patient-level and system-level barriers shape real-world diagnostic practices. The use of phenotype-specific ICD-10 codes across a large national sample indicates that some diagnostic processes may be occurring but are not adequately captured in administrative data.

Addressing these diagnostic inequities is essential to improving HF care. Given that accurate HF phenotyping is essential for appropriate therapeutic selection, these diagnostic disparities may contribute to suboptimal treatment outcomes and persistent health inequities in cardiovascular care. Policy reforms that expand access to imaging - through strategies such as mobile echocardiography units, telemedicine infrastructure, or reimbursement parity - could reduce structural gaps. Provider-facing interventions like guideline adherence training, decision support tools, and standardized diagnostic protocols may also enhance the accuracy and consistency of HF diagnosis, especially in under-resourced settings. Future research should explore how disparities in diagnostic access affect clinical outcomes and assess the effectiveness of targeted solutions. Ultimately, ensuring equitable access to diagnostic tools is a critical step toward achieving better, more timely care for all patients with HF.
